# Knowledge, attitude, and practice toward stroke among university students in Saudi Arabia: a nationwide cross-sectional study

**DOI:** 10.3389/fpubh.2026.1742770

**Published:** 2026-02-13

**Authors:** Mothana Al Jaber, Hassan Khafaji, Saleh Alanazi, Fay Hadi, Rena Abualjamal, Abdulelah Almasaud, Abrar Alzahrani, Suliman Alluhib, Mayar Alzain, Saud Al Naim

**Affiliations:** 1 College of Medicine, King Faisal University, Al-Ahsa, Saudi Arabia; 2 College of Medicine, King Abdulaziz University, Jeddah, Saudi Arabia; 3 College of Medicine, Prince Sattam University, Al-Kharj, Saudi Arabia; 4 College of Medicine, Jeddah University, Jeddah, Saudi Arabia; 5 College of Medicine, King Saud bin Abdulaziz University for Health Sciences, Jeddah, Saudi Arabia; 6 College of Medicine, Qassim University, Buraydah, Saudi Arabia; 7 College of Medicine, Taif University, Taif, Saudi Arabia; 8 Department of Neurology, College of Medicine, King Faisal University, Al-Ahsa, Saudi Arabia

**Keywords:** attitude, knowledge, medical students, Saudi Arabia, stroke

## Abstract

**Introduction:**

Stroke remains to be a primary cause of mortality and disability globally, imposing a significant economic burden. The incidence and fatality rates of stroke in Saudi Arabia have risen, potentially due to inadequate public awareness. University students represent a crucial group for targeted education and community-based preventive programs. This study sought to evaluate the knowledge, attitudes, and practices (KAP) on stroke among university students in Saudi Arabia and to identify parameters related to stroke literacy to guide future national awareness initiatives.

**Methods:**

This nationwide cross-sectional study was conducted between January 5 and May 10, 2025, among undergraduate students from public and private universities located in the Eastern, Western, Central, and Southern regions of Saudi Arabia. Data was collected both online and in person using a validated, self-administered questionnaire assessing demographic characteristics, knowledge of stroke symptoms and risk factors, and attitudes and preventive practices. A total knowledge score (0–14) was computed, and univariate and multivariate linear regression analyses were performed to identify predictors of stroke knowledge.

**Results:**

A total of 484 students participated (mean age 21.9 ± 4.1 years; 52.7% female). Health-related students constituted 41.7% of the sample. Overall, 77.9% had prior awareness of stroke, mainly from schools (34.1%) and the internet (32.9%). The mean knowledge score was 9.17 ± 2.8, reflecting moderate awareness. High blood pressure was the most recognized risk factor (85.1%), and neurological effects were the most identified consequence (73.8%). In multivariate analysis, female sex, health-related specialty, senior academic level, and personal or family experience with stroke were significant predictors of higher knowledge scores (*p* < 0.05).

**Conclusion:**

This study provides the first nationwide, multi-university assessment of stroke awareness among Saudi university students. Findings highlight moderate knowledge but inadequate preventive practices, emphasizing the need for structured educational programs and campus-based stroke literacy campaigns to improve early recognition and timely response across the country.

## 1 Introduction

Stroke is a leading cause of death and long-term disability worldwide. Each year, more than 12 million people experience a stroke and about 6.6 million die from its complications (1). In the Global Burden of Disease (GBD 2021) study, stroke accounted for roughly 143 million disability-adjusted life years (DALYs), underscoring its profound social and economic impact as well as the substantial costs of acute care, rehabilitation, and long-term support for survivors (1–3).

Two major subtypes predominate; ischemic stroke, caused by arterial occlusion, and hemorrhagic stroke, due to vessel rupture and intracerebral bleeding (3). Risk is driven by modifiable factors, mainly hypertension, diabetes, dyslipidemia, obesity, atrial fibrillation, smoking, unhealthy diet, and physical inactivity, and by non-modifiable factors such as age, sex, and family history (4, 5).

In Saudi Arabia, the burden remains considerable. The estimated annual incidence is ~29 per 100,000, with in-hospital mortality reported between 8 and 28%, although some regions have shown modest recent improvements (6–8). Public awareness, however, is limited. In a Riyadh survey, many respondents recognized hypertension as a key cause, yet few could identify warning signs or the appropriate emergency response (9).

Existing research on stroke awareness in Saudi Arabia has largely been confined to single universities or individual cities, often focusing on medical students. To date, no large-scale study has evaluated stroke-related knowledge, attitudes, and practices (KAP) among university students across multiple regions. This gap matters as most strokes are preventable through early recognition and control of modifiable risks, and establishing healthy behaviors during university years can reduce future cardiovascular burden (10–12). Prior evidence suggests that addressing core behavioral and metabolic risk factors, especially blood-pressure control, smoking cessation, and regular physical activity, could prevent approximately 80–90% of stroke cases globally (7, 10).

Accordingly, the present study evaluates KAP related to stroke among university students in Saudi Arabia and identifies factors associated with better awareness. The findings are intended to inform practical, scalable educational interventions that promote prevention, early recognition, and timely response at a national level.

## 2 Materials and methods

### 2.1 Study design

**Figure 1:**
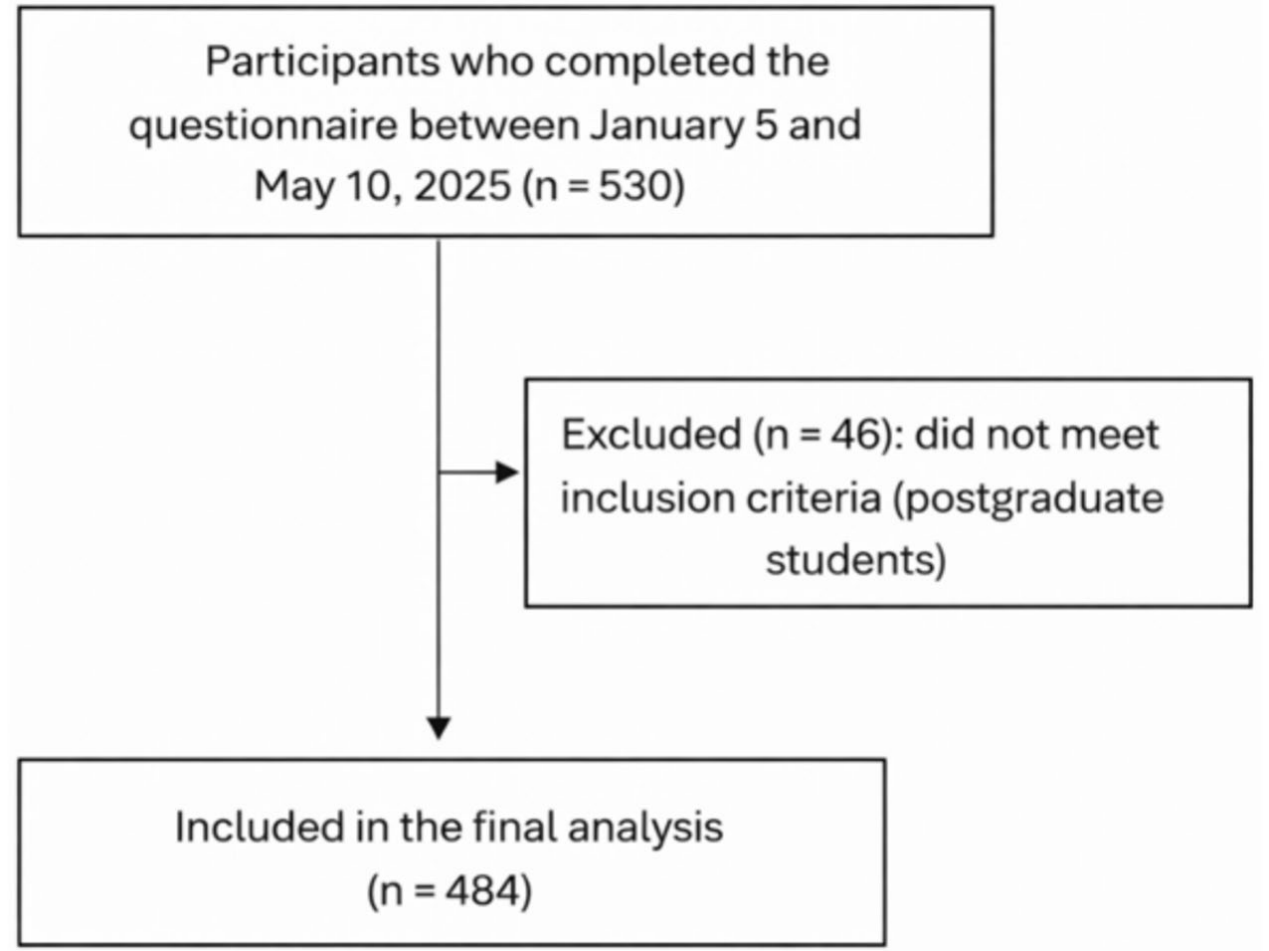
Flow chart for the selection process.

This nationwide cross-sectional study was conducted between January 5 and May 10, 2025, among undergraduate students (*n* = 484) enrolled at public and private universities across Saudi Arabia (Figure 1). Participating institutions represented the four major geographic regions of the country: Central, Eastern, Western, and Southern region. This wide institutional distribution ensured adequate national representation.

Ethical approval was obtained from the Institutional Review Board of King Faisal University (Approval No. KFU-REC-2025-JAN-ETHICS3006). All participants provided informed consent electronically or in person before participation. Confidentiality and anonymity were maintained throughout the study.

### 2.2 Study population and sample size

The study population included undergraduate students enrolled in Saudi universities during the 2024–2025 academic year. Eligible participants were students from any level or discipline who met the following inclusion criteria:

- Aged 18 years or older.
- Currently enrolled in a public or private Saudi university.
- Able and willing to provide informed consent and complete the questionnaire in Arabic or English.

Students who did not meet the inclusion criteria provided incomplete responses, or submitted duplicate entries were excluded from the analysis.

The required sample size was calculated using a single population proportion formula, assuming a 95% confidence interval, 50% expected proportion (as no prior national data were available), and a 5% margin of error. The minimum required sample size was 385 participants. To account for potential non-responses and exclusions, an additional 25% was added, yielding a target sample of approximately 480 students. A total of 484 valid responses were ultimately included in the final analysis, exceeding the calculated requirement and ensuring adequate statistical power.

### 2.3 Sampling strategy

A convenience sampling strategy was used to recruit participants from universities across different regions of Saudi Arabia. Recruitment was conducted through three complementary channels to ensure broad coverage and diversity of disciplines.

In-person recruitment was carried out by members of the research team, who approached students on university campuses in shared areas such as libraries, cafeterias, and student activity centers, inviting them to participate voluntarily. Online dissemination was also conducted through social media platforms, including WhatsApp and X (formerly Twitter), accompanied by a brief description of the study purpose and eligibility criteria. In addition, collaborating faculty members and student affairs offices at participating universities distributed the official invitation and questionnaire link via university email systems to reach a wider audience. The invitation letter provided information about the study objectives, confidentiality assurances, and estimated completion time. Participation was entirely voluntary, and informed consent was obtained electronically before respondents could proceed to the questionnaire. To maintain data integrity, each participant was allowed to submit the survey only once.

To assess the representativeness of the study sample, the distribution of key demographic characteristics (including sex, region, and type of university [public vs. private]) was compared descriptively with publicly available national statistics on undergraduate enrollment in Saudi Arabia. Overall, the sample demonstrated a distribution broadly comparable to national university enrollment patterns, although some differences are expected due to the use of convenience sampling.

### 2.4 Data collection

Data were collected using a self-administered questionnaire distributed through multiple channels, including online distribution via Google Forms, in-person invitations on university campuses, and official university-wide email circulation coordinated through the Deanships of Information Technology and Scientific Research. The authors of the study represent a collaborative research team from universities across different regions of the country, including the Eastern, Western, Central, and Southern regions, to ensure broad geographic coverage.

We assessed student knowledge, attitudes, and practices toward stroke. The questionnaire was developed based on previously published tools assessing stroke awareness (13–17), with most items adapted from previously published Arabic-language questionnaires to ensure cultural and contextual relevance. Additional items were adapted from a validated survey conducted among university students in Jazan, Saudi Arabia (18). The adaptation process involved reviewing all candidate items for relevance to the study objectives, clarity, and appropriateness for undergraduate students, with minor wording modifications made where necessary without altering the original meaning of the items.

Content validity was assessed by a panel of experts consisting of public health specialists and clinical researchers with experience in stroke research, who evaluated the questionnaire for relevance, clarity, and completeness. Feedback from the panel was incorporated into the final version of the instrument. As most items were derived from previously validated Arabic instruments, the questionnaire was administered in Arabic. No forward–backward translation was required. The final Arabic version was reviewed to ensure linguistic clarity and consistency across items.

The instrument included 14 knowledge-based questions covering stroke warning signs, risk factors, affected organs, and appropriate response behaviors. Each correct response received one point, while incorrect answers received zero points. The total stroke knowledge score thus ranged from 0 to 14, with higher scores indicating greater knowledge. To assess reliability, a pilot test was conducted with 50 university students, and internal consistency of the knowledge domain was evaluated using Cronbach’s alpha, which yielded a value of 0.87, indicating good reliability.

### 2.5 Statistical analysis

Data were analyzed using IBM SPSS Statistics version 26 (IBM Corp, Armonk, NY). Descriptive statistics, including means, standard deviations, frequencies, and percentages, were used to summarize demographic data and knowledge scores. For inferential analysis, univariate linear regression was performed to explore associations between demographic variables and stroke knowledge scores. Variables that showed significant associations in the univariate model (*p* < 0.05) were entered into a multivariate linear regression model to identify independent predictors of stroke knowledge. Regression results were reported using *β* coefficients, *p*-values, and 95% CIs. Prior to model interpretation, assumptions of linear regression were evaluated. Normality of residuals was assessed using visual inspection of histograms and normal probability (Q–Q) plots, while homoscedasticity was examined through residual-versus-fitted value plots. No substantial deviations from model assumptions were observed. A *p*-value of < 0.05 was considered statistically significant.

## 3 Results

### 3.1 Participant characteristics

**Figure 2:**
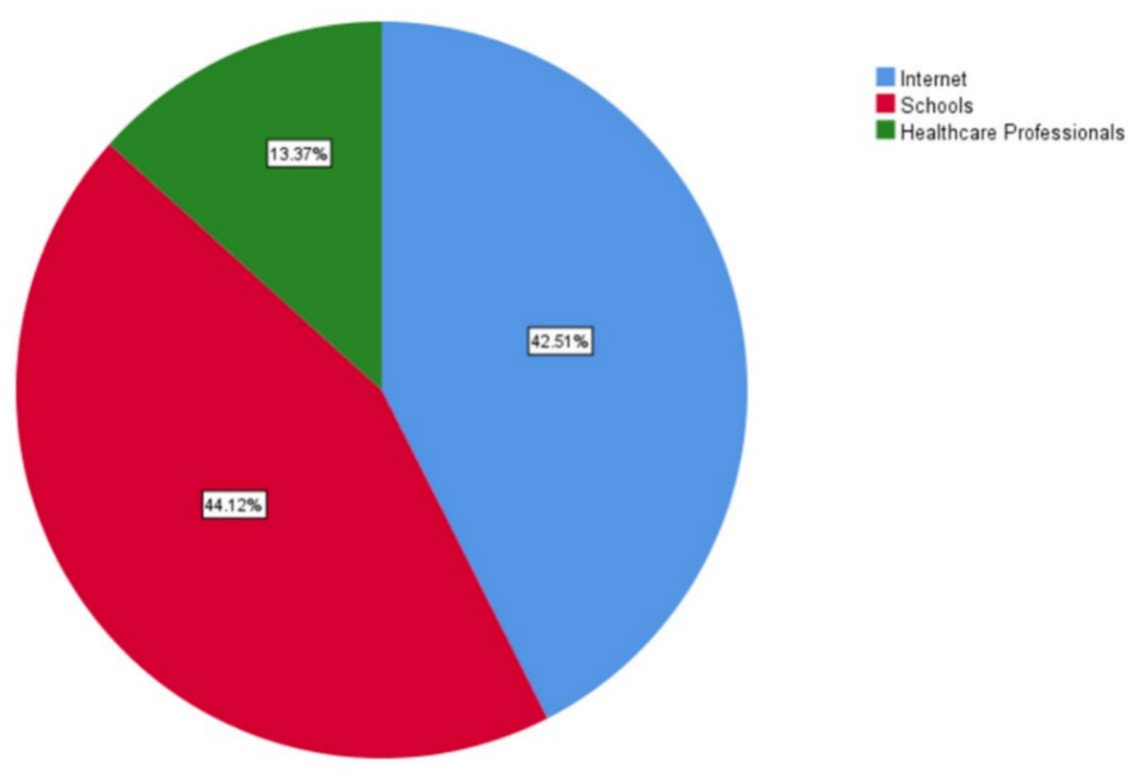
Cerebrovascular accident knowledge resources.

The final sample consisted of 484 university students from across Saudi Arabia. Of these, 52.7% (*n* = 255) were female and 47.3% (*n* = 229) were male, with a mean age of 21.89 years (SD = 4.1). Most participants were single (95.2%, *n* = 461), while 4.8% (*n* = 23) were married. Regarding academic specialization, 41.7% (*n* = 202) were enrolled in health-related disciplines, and 58.3% (*n* = 282) were in non-health fields. The majority of respondents (87.4%, *n* = 423) reported no chronic illnesses, whereas 12.6% (*n* = 61) reported having at least one chronic condition. In terms of academic level, 28.1% (*n* = 136) were fourth-year students, followed by 20.2% (*n* = 98) in the third year, 19.0% (*n* = 92) in the second year, 12.6% (*n* = 61) in the fifth year, 10.3% (*n* = 50) in the sixth year, and 9.7% (*n* = 47) in the first year. Regarding the geographical distribution of university enrollment, the majority of participants were registered in Universites located in the central area (36.4%, *n* = 176), followed by the western region (26.4%, *n* = 128) and the eastern region (25.2%, *n* = 122). The remaining participants were registered at universities in the southern region (12.0%, *n* = 58; Figure 2).

Prior knowledge of cerebrovascular accidents (strokes) was reported by 77.9% (*n* = 377) of participants. Regarding exposure, 16.5% (*n* = 80) indicated having relatives who had suffered a stroke, while 27.9% (*n* = 135) personally knew someone who had experienced a stroke.

Sources of prior stroke knowledge varied: schools (34.1%, *n* = 165) and the internet (32.9%, *n* = 159) were the most common, followed by healthcare professionals (10.3%, *n* = 50), while the remainder cited friends, social media, or other sources (Table 1).

**Table 1:** Characteristics of the participants.

Variable	n	%
Age, years, mean ± SD	21.89 ± 4.1	
Sex		
Female	255	52.7
Male	229	47.3
Marital status		
Single	461	95.2
Married	23	4.8
Specialty		
Health	202	41.7
Non-health	282	58.3
Chronic diseases		
No	423	87.4
Yes	61	12.6
Level		
First year	47	9.7
Second year	92	19
Third year	98	20.2
Fourth year	136	28.1
Fifth year	61	12.6
Sixth year	50	10.3
Region of University		
Eastern	122	25.2
Central	176	36.4
Western	128	26.4
Southern	58	12.0
Previous Knowledge about CVA		
No	107	22.1
Yes	377	77.9
Do you have relatives who have had a stroke?		
No	404	83.5
Yes	80	16.5
Do you know someone who has had a stroke?		
No	349	72.1
Yes	135	27.9

CVA, cerebrovascular accident.

### 3.2 Knowledge score

**Figure 3:**
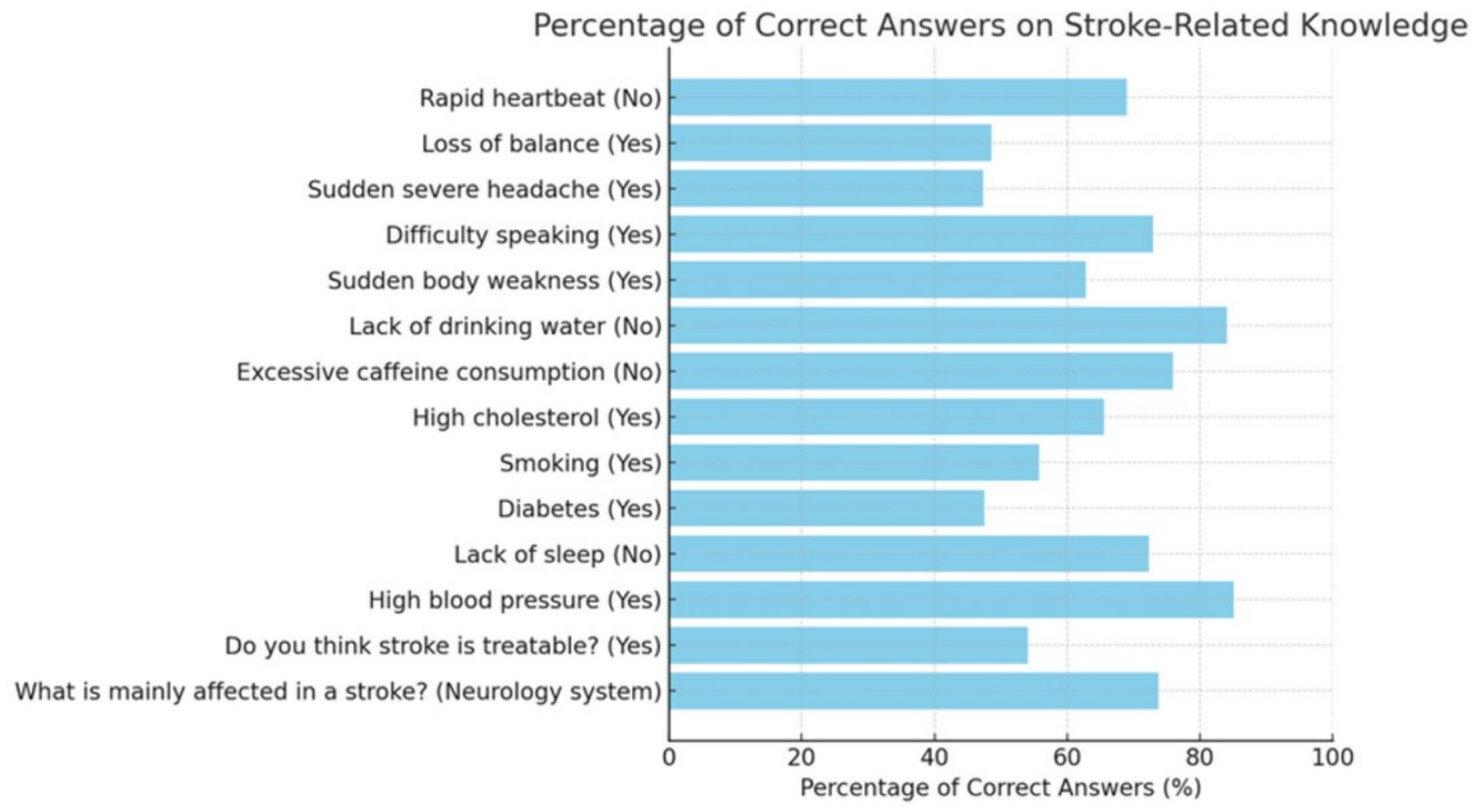
Percentage of correct answers in stroke-related knowledge.

The effect of stroke most recognized by participants was its impact on the neurological system, identified correctly by 73.8% of respondents (mean = 0.74, SD = 0.44; Figure 3). In contrast, only 54.1% (mean = 0.54, SD = 0.49) were aware that stroke is a treatable condition. Among the listed risk factors, high blood pressure was the most frequently recognized (85.1%, mean = 0.85, SD = 0.35), whereas diabetes was the least identified (47.5%, mean = 0.48, SD = 0.50). Regarding warning signs, difficulty in speaking and sudden body weakness were the most commonly known, with correct responses from 72.9 and 62.8% of participants, respectively. The mean overall knowledge score was 9.17 (SD = 2.8) out of 14, reflecting a moderate level of awareness about stroke risk factors, symptoms, and outcomes (Table 2).

**Table 2:** Knowledge score.

Variable	Correct answers (n, %)	Mean (SD)
What is mainly affected in a stroke? (Neurology system)	357, 73.8%	0.74 (0.44)
Do you think stroke is treatable? (Yes)	262, 54.1%	0.54 (0.49)
Risk factors		
High blood pressure (Yes)	412, 85.1%	0.85 (0.35)
Lack of sleep (No)	350, 72.3%	0.72 (0.44)
Diabetes (Yes)	230, 47.5%	0.48 (0.50)
Smoking (Yes)	270, 55.8%	0.56 (0.49)
High cholesterol (Yes)	317, 65.5%	0.65 (0.47)
Excessive caffeine consumption (No)	368, 76%	0.76 (0.42)
Lack of drinking water (No)	407, 84.1%	0.84 (0.36)
Warning signs		
Sudden body weakness (Yes)	304, 62.8%	0.63 (0.48)
Difficulty speaking (Yes)	353, 72.9%	0.73 (0.44)
Sudden, severe headache (Yes)	229, 47.3%	0.47 (0.5)
Loss of balance (Yes)	235, 48.6%	0.49 (0.5)
Rapid heartbeat (No)	334, 69%	0.69 (0.46)
Total knowledge score		9.17 (2.8)

Mean stroke knowledge scores showed a graded increase with advancing year of study. Lower scores were observed among first- and second-year students (8.06 ± 2.34 and 7.76 ± 2.37, respectively), while progressively higher scores were noted among students in the fourth to sixth years of study (9.60 ± 2.84, 10.26 ± 2.76, and 11.50 ± 2.51, respectively), indicating a positive association between academic level and stroke knowledge (Table 3).

**Table 3:** Knowledge score by study year.

Year of study	n	Mean knowledge score (SD)
1st year	47	8.06 (2.34)
2nd year	92	7.76 (2.37)
3rd year	98	8.52 (2.34)
4th year	136	9.60 (2.84)
5th year	61	10.26 (2.76)
6th year	50	11.50 (2.51)

### 3.3 Regression analysis

In the univariate analysis, male participants demonstrated significantly lower knowledge scores (*β* = −1.194, *p* < 0.001), whereas students enrolled in health-related disciplines scored higher (*β* = 2.922, p < 0.001). Having a relative or acquaintance who had experienced a stroke was also associated with higher knowledge levels (*β* = 2.113, *p* < 0.001 and *β* = 1.612, *p* < 0.001, respectively). Educational level showed a positive effect, with students in their fourth to sixth years of study demonstrating higher awareness (*β* = 2.003, *p* < 0.001).

In the multivariate model, these associations remained largely consistent. Male sex continued to be associated with lower scores (*β* = −0.601, *p* = 0.006), while being in a health-related specialty predicted higher knowledge (*β* = 2.06, *p* < 0.001). Having relatives who had suffered a stroke also remained a significant predictor (*β* = 1.096, *p* = 0.002), whereas the effect of personally knowing someone with a stroke became less pronounced (*β* = 0.642, *p* = 0.03). These results indicate that academic background, personal exposure, and gender differences play key roles in determining stroke awareness among university students (Table 4).

**Table 4:** Regression analysis.

Univariate				
Variable	β	Lower 95%CI	Upper 95%CI	*p*-value
Age: >20 years	−0.001	−0.061	0.06	0.978
Sex: Male	−1.194	−1.685	−0.703	<0.001
Marital status: Married	−1.02	−2.197	0.158	0.08
Specialty: Health	2.922	2.486	3.359	<0.001
Chronic diseases: Yes	−0.414	−1.17	0.342	0.282
Level: 4th year to 6th year	2.003	1.534	2.472	<0.001
Previous Knowledge about CVA: Yes	2.027	1.449	2.604	<0.001
Do you have relatives who have had a stroke? Yes	2.113	1.464	2.762	<0.001
Do you know someone who has had a stroke? Yes	1.612	1.071	2.153	<0.001
Multivariate				
Sex: Male	−0.601	−1.031	−0.172	0.006
Specialty: Health	2.06	1.599	2.521	<0.001
Level: 4th year to 6th year	1.001	0.576	1.426	<0.001
Previous Knowledge about CVA: Yes	0.939	0.429	1.45	<0.001
Do you have relatives who have had a stroke? Yes	1.096	0.41	1.782	0.002
Do you know someone who has had a stroke? Yes	0.642	0.059	1.226	0.03

CVA, cerebrovascular accident.

### 3.4 Public perceptions and actions on stroke prevention and response

Most respondents (90.9%) believed that stroke is a preventable condition. When asked about the importance of maintaining a healthy lifestyle, 82.6% considered it “very important,” and 13.8% regarded it as “important.” A strong majority (88.8%) agreed that individuals experiencing stroke symptoms should seek immediate medical care. Regular health checkups were perceived as “very important” by 68.2% of participants, although 6.4% felt they could be ignored if a person appeared healthy. Nearly half of the respondents (49.8%) reported that they had never advised others on stroke prevention, and 33.3% stated that they lacked adequate knowledge to do so. Only 14.3% reported monitoring their own health indicators, such as blood pressure or cholesterol, on a regular basis, whereas 49.6% did so occasionally. When presented with a stroke scenario, 87.2% of students indicated that they would seek immediate medical assistance, reflecting generally positive attitudes toward emergency response (Table 5).

**Table 5:** Public awareness and practices regarding stroke prevention and immediate response.

Variable	n	%
Do you think strokes are preventable?		
No	17	3.5
Not sure	27	5.6
Yes	440	90.9
How important is a healthy lifestyle to prevent stroke?		
Not important	8	1.7
Not sure	9	1.9
Important	67	13.8
Very important	400	82.6
In your opinion, should a person experiencing symptoms of a stroke seek medical care immediately?		
Agree	24	5
Strongly agree	430	88.8
Disagree	1	0.2
Neutral	4	0.8
Depends on severity of symptoms	25	5.2
How important are regular health checkups to prevent stroke?		
Not important	3	0.6
Not sure	12	2.5
Important	108	22.3
Very important	330	68.2
Can be ignored if you feel well	31	6.4
Have you ever given advice to someone about preventing or dealing with a stroke?		
No	241	49.8
I do not have enough information	161	33.3
Yes	82	16.9
Are you monitoring your health (such as blood pressure or cholesterol levels)?		
Sometimes	240	49.6
Regularly	69	14.3
Never	175	36.2
If you or someone you know is experiencing symptoms of a stroke, what would you do?		
Give the patient medication available at home	9	1.9
Wait to see if symptoms resolve	21	4.3
Wait for symptoms to improve, then transfer to the hospital	16	3.3
Seek immediate medical help	422	87.2
I am not sure	16	3.3

## 4 Discussion

This nationwide study evaluated the knowledge, attitudes, and practices toward stroke among university students in Saudi Arabia. Overall, the findings show that, although most participants had heard of stroke and expressed favorable attitudes toward prevention, substantial and clinically relevant knowledge gaps persist regarding stroke risk factors, early warning signs, and appropriate emergency response. The observed mean knowledge score of 9.17/14 indicates a moderate level of awareness, consistent with earlier Saudi and international studies (4, 13–17). Similar levels of knowledge were reported by Al-Otaibi et al. in Riyadh and by Rizvi et al. at Majmaah University, where students were generally able to identify common symptoms but showed incomplete understanding of risk profiles and comprehensive prevention (14, 15). These concordant results suggest that, despite wider access to digital health information, stroke literacy among young adults remains suboptimal.

As expected, students in health-related colleges scored significantly higher than those in non-health programs, a pattern repeatedly documented in Saudi and regional literature (14, 15, 17). This likely reflects structured exposure to cardiovascular and neurological content, clinical reasoning, and emphasis on time-sensitive emergencies in health curricula, whereas non-health students depend mainly on informal or non-systematic sources such as social media. Likewise, senior (higher-year) students demonstrated better awareness than juniors, mirroring the findings of Almalki et al. at King Faisal University and reinforcing the role of cumulative educational exposure in shaping health literacy (15). These findings underscore that field of study and academic level are key, modifiable touchpoints through which universities can influence stroke-related knowledge.

Gender-based differences were also apparent, with female students showing higher awareness than males, in line with previous local and regional work (10, 14). Possible explanations include greater health-seeking behavior, higher engagement with preventive-health content, and greater responsiveness to health campaigns among females. In addition, participants who reported a personal or family history of stroke achieved higher knowledge scores, which is consistent with observations from Jazan and Lebanon (13, 18). This highlights the value of experiential and vicarious learning, as exposure to real cases appears to sensitize individuals to symptomatology, seriousness, and the importance of rapid response.

Despite these encouraging patterns, several critical gaps were identified. Fewer than half of the students recognized diabetes and smoking as stroke risk factors, and less than 50% identified less “classic” warning signs such as sudden severe headache, vertigo, or imbalance. Comparable gaps were described by Rizvi et al. and by Sowtali et al. in Malaysia, where students tended to recall only the most visible symptoms (e.g., hemiparesis, facial droop, or aphasia) but not the full clinical spectrum (7, 14). This under-recognition is concerning in the Saudi context, where metabolic risk factors, sedentary lifestyle, and smoking (including e-cigarettes) are increasingly prevalent in young adulthood. Addressing these modifiable risks early could substantially reduce downstream cardiovascular and cerebrovascular burden at the population level. Accordingly, university-level health promotion should give greater weight to “silent” or underappreciated risks represented in diabetes, dyslipidemia, central obesity, and tobacco use, and explicitly link them to stroke and other vascular outcomes.

To bridge these gaps, integrating brief, competency-oriented stroke-awareness modules into university orientation programs, common health-education courses, or service-learning activities would be beneficial. Encouraging student participation in campus-based campaigns, peer-to-peer education, and community outreach can further extend the impact of university initiatives beyond campus and into households. Similar strategies have been shown to improve health literacy and intention to seek urgent care in other Gulf and regional settings (14, 17). In the present study, schools and the internet were the leading sources of information, whereas healthcare professionals were infrequently cited, exactly the pattern reported in Riyadh and the Eastern Province (13, 17). This reliance on digital and informal channels underscores the importance of producing credible, visually appealing, and culturally tailored stroke content for social media, but with oversight from health professionals. Recent digital campaigns and short educational videos in the region have demonstrated promising increases in recognition of stroke warning signs and willingness to call emergency services (19, 20).

An important behavioral finding is the mismatch between positive attitudes and limited preventive practice. Similar attitude–practice gaps have been reported among university populations in neighboring countries and in non-medical colleges, indicating that knowledge alone is insufficient to trigger action (21, 22). Incorporating these components into university health programs, e.g., BP screening days, smoking-cessation challenges, step-count competitions, or simulation drills using FAST/BE-FAST, could help convert intention into behavior and improve timely help-seeking (23). Universities are well positioned to host such activities and to use students as “health ambassadors,” an approach that has improved preventive behaviors in other campus-based interventions (24). Behavior-change frameworks such as the Health Belief Model emphasize constructs that were not directly targeted in most existing awareness activities, perceived susceptibility, perceived benefits, self-efficacy, and actionable cues (25). Embedding stroke content in general-education requirements and linking it to practical simulations (“recognize-and-respond”) would further reinforce learning and emergency readiness (19, 20, 26).

Situating our findings within current global evidence strengthens their public-health relevance. The GBD 2021 stroke analysis (reported in 2024) confirms that stroke remains a top cause of death and disability worldwide and that most of the burden is attributable to modifiable metabolic and behavioral risk factors (22). Likewise, the 2024 AHA/ASA primary prevention recommendations call for a life-course, “start early” strategy that targets young adults to reduce first-ever stroke through integrated vascular-risk management, healthier lifestyles, and rapid symptom recognition (19). Our data identify exactly where Saudi university students fall short, recognizing less obvious symptoms, appreciating metabolic risks, and translating awareness into practice; thereby providing actionable targets for national and campus-level interventions.

Prior Saudi studies in university populations have largely been single-site and/or limited to specific regions or student subgroups. For example, an analytic cross-sectional study among Jazan University students documented gaps in recognition of stroke risk factors and symptoms despite broad awareness of the condition (18). Similarly, work among undergraduate health-care students in Riyadh reported variability in knowledge and persistent deficits related to risk factors and warning signs (27). Another study conducted at Northern Border University assessed students’ awareness of stroke and identified gaps in knowledge regarding risk factors and warning signs, but its findings were restricted to a single region and did not comprehensively address attitudes and practices (28). In contrast, the present study extends the existing literature by adopting a nationwide, multi-regional approach and assessing knowledge, attitudes, and practices toward stroke among undergraduate students. By recruiting participants from public and private universities across the major regions of Saudi Arabia, our study provides a broader and more comprehensive national perspective on stroke awareness in young adults, thereby addressing important gaps left by earlier single-site investigations.

### 4.1 Strengths and limitations

The main strength of this study is its extensive, multi-regional sample, which provides a broad national overview of stroke awareness among Saudi university students. Using a validated questionnaire with good internal consistency (Cronbach’s alpha = 0.87) enhances the reliability and comparability of the results. Nevertheless, this study’s convenience sampling and online distribution may over-represent digitally engaged or health-interested students, limiting generalizability across regions, institution types, and non-university youth. Its cross-sectional design precludes causal inference, and self-reported knowledge/practices are vulnerable to recall and social-desirability biases. Although internal consistency was good, item content may overweight “visible” signs over subtler symptoms, risking misclassification. Unmeasured confounding (e.g., prior coursework, family health literacy), unmodeled clustering by college/university, potential nonresponse bias, and timing effects (e.g., proximity to exams or campaigns) may further influence estimates. Heavy reliance on rapidly changing digital information environments and unassessed accessibility needs (e.g., rural campuses, sensory impairments) could also mask equity gaps in stroke literacy. Also, as this study sampled the general university population, the availability of detailed subgroup-specific documentation was limited; however, the findings offer a useful baseline to inform future targeted studies in more narrowly defined student cohorts.

### 4.2 Implications and recommendations

Universities should integrate brief, competency-based stroke awareness modules into student orientation and general health courses, reinforced through periodic refreshers and practical FAST/BE-FAST simulations to strengthen early recognition and emergency response (20). Preventive initiatives should prioritize under-recognized vascular risk factors (e.g., diabetes, dyslipidemia, tobacco and e-cigarette use) and target non-health disciplines, early-year students, and males through peer-led programs and clinician-guided digital content. These interventions should be grounded in behavior-change frameworks such as the Health Belief Model and aligned with national noncommunicable disease prevention priorities under Saudi Vision 2030 to ensure scalability and sustainability.

## 5 Conclusion

Our findings revealed a moderate level of stroke awareness among university students in Saudi Arabia, alongside notable deficits in recognizing key risk factors and early warning signs. Health-related majors, female students, seniors, and those with personal exposure to stroke demonstrated higher knowledge, whereas males and non-health students scored lower. Although attitudes toward prevention were positive, preventive practices lagged behind. These results highlight the need for targeted, behaviorally informed educational interventions to improve stroke literacy, strengthen emergency response intentions, and ultimately reduce the future stroke burden in this young population.

## Data Availability

The raw data supporting the conclusions of this article will be made available by the authors, without undue reservation.
